# Automated Cyber and Privacy Risk Management Toolkit

**DOI:** 10.3390/s21165493

**Published:** 2021-08-15

**Authors:** Gustavo Gonzalez-Granadillo, Sofia Anna Menesidou, Dimitrios Papamartzivanos, Ramon Romeu, Diana Navarro-Llobet, Caxton Okoh, Sokratis Nifakos, Christos Xenakis, Emmanouil Panaousis

**Affiliations:** 1 ATOS Spain, Atos Research & Innovation, Cybersecurity Unit, 08020 Barcelona, Spain; 2 UBITECH Ltd., Thessalias 8 & Etolias 10, 152 31 Chalandri, Greece; smenesidou@ubitech.eu (S.A.M.); dpapamartz@ubitech.eu (D.P.); 3 Fundació Privada Hospital Asil de Granollers, 08402 Granollers, Spain; rromeu@fhag.es (R.R.); diananavarro@fphag.org (D.N.-L.); 4 School of Computing and Mathematical Sciences, University of Greenwich, London SE10 9LS, UK; c.okoh@greenwich.ac.uk (C.O.); e.panaousis@greenwich.ac.uk (E.P.); 5 Karolinska Institutet Department of Learning, Informatics, Management and Ethics, Tomtebodavägen 18b, 171 77 Solna, Sweden; sokratis.nifakos@ki.se; 6 Department of Digital Systems, University of Piraeus, Karaoli ke Dimitriou 80, 185 34 Pireas, Greece; xenakis@unipi.gr

**Keywords:** toolkit, cybersecurity, privacy, risk assessment, risk control, healthcare

## Abstract

Addressing cyber and privacy risks has never been more critical for organisations. While a number of risk assessment methodologies and software tools are available, it is most often the case that one must, at least, integrate them into a holistic approach that combines several appropriate risk sources as input to risk mitigation tools. In addition, cyber risk assessment primarily investigates cyber risks as the consequence of vulnerabilities and threats that threaten assets of the investigated infrastructure. In fact, cyber risk assessment is decoupled from privacy impact assessment, which aims to detect privacy-specific threats and assess the degree of compliance with data protection legislation. Furthermore, a Privacy Impact Assessment (PIA) is conducted in a proactive manner during the design phase of a system, combining processing activities and their inter-dependencies with assets, vulnerabilities, real-time threats and Personally Identifiable Information (PII) that may occur during the dynamic life-cycle of systems. In this paper, we propose a cyber and privacy risk management toolkit, called AMBIENT (Automated Cyber and Privacy Risk Management Toolkit) that addresses the above challenges by implementing and integrating three distinct software tools. AMBIENT not only assesses cyber and privacy risks in a thorough and automated manner but it also offers decision-support capabilities, to recommend optimal safeguards using the well-known repository of the Center for Internet Security (CIS) Controls. To the best of our knowledge, AMBIENT is the first toolkit in the academic literature that brings together the aforementioned capabilities. To demonstrate its use, we have created a case scenario based on information about cyber attacks we have received from a healthcare organisation, as a reference sector that faces critical cyber and privacy threats.

## 1. Introduction

Cyber Risk Management has traditionally been a fundamental challenge of every organisation that seeks ways to protect its assets against cyber threats [1]. This is about using cybersecurity countermeasures (technical, operational, and physical) to prevent, detect, and respond to cyber attacks prohibiting the exploitation of the organisation. Technical controls can be anything from “Inventory and Control of Hardware Assets” to “ Penetration Tests and Red Team Exercises”, according to the Center for Internet Security (CIS) Controls [2]. Operational controls refer to standards, policies, and frameworks adopted by the organisation while physical security measures can prevent physical access to the cyber infrastructure.

In most cases, implementing all controls is neither possible nor required although some controls are necessary for an organisation to operate. For example, corporate cybersecurity strategies dictate the need for aligning with information security frameworks such as the International Organization for Standardization (ISO (https://www.iso.org/isoiec-27001-information-security.html (accessed on 14 June2021))) 27001/2. Regarding the different types of organisations, the National Institute of Standards and Technology (NIST) has published the Framework for Improving Critical Infrastructure Cybersecurity stating that different organisations exhibit different cyber risks due to their different security requirements and infrastructures to be protected. For instance, financial and healthcare organisations have regulatory requirements to satisfy, while the second have also to protect human lives [3].

Our work is motivated by the need to undertake cyber risk management in the healthcare domain. Nevertheless, cyber risk management methodologies and tools are generally applicable to a variety of industries with the underlying models and system components to remain the same. Our choice was initially motivated by the criticality of this domain determined by cyber–physical impact inflicted by cyber risks as human lives can be at risk following a cyber incident. In the recent 2020 Data Breach Investigations Report, published by Verizon, Healthcare is listed as the industry with the majority of data breaches [4].

Last year the same report indicated that healthcare stands out due to the fact that 59% of breaches are associated with internal actors, and 81% of the incidents within Healthcare corresponds to miscellaneous errors, privilege misuse and web applications. Only in the United States, healthcare organisations have reported since 2016 over 170 individual ransomware attacks, affecting around 1500 healthcare centres and over 6.5 million patients, which represents an estimated cost to the industry of USD 157 million [5]. This rising number of security incidents has also led to data breaches (e.g., 72% of the data breaches were medical [6]), due to the massive amount of sensitive data that is processed. The observations worsen if we look at the currently overwhelmed healthcare domain due to the COVID-19 pandemic.

The well-known WannaCry ransomware, although not targeting healthcare organisations per se, managed to massively affect the UK’s National Healthcare System (NHS) posing not only financial damages, but also life-threatening ones, for example, via operations which could not take place when systems went down during the attack [7]. The low security posture of many hospitals was the reason for WannaCry exploiting so successfully the various hosts causing tremendous impact during a limited period of time. Having a state actor behind this attack is not the only source of danger to healthcare organisations, which may also be susceptible to attacks launched by anyone ranging from script kiddies through to organised crime and state actors.

Besides, healthcare data are more valuable than many other data in the Dark Web, because of the potential adversarial use of it, including blackmailing to gain some financial gain, sell intelligence to pharmaceutical companies, as well as compromising data integrity to create chaos in a country or a hospital such as the recent incident at Valley Hospital, in California, which was hit by a ransomware attack on 11 October 2020. The case of WannaCry clearly demonstrated that UK hospitals had not invested in cyber security controls while post-incident analysis (https://www.digitalhealth.net/2019/08/nhs-trusts-it-spend-up-more-than-150m-since-wannacry/ (accessed on 10 May 2021)) shows that 65 NHS Trusts spent 612 million pounds on IT two years after the attack took place, which corresponds to 33% budget increase compared to the year preceding this incident.

While everything shows that cybersecurity has been more of an afterthought for healthcare organisations than, for instance, for the banking industry, it is also clear that due to the General Data Protection Regulation (GDPR) [8], hospitals are obliged to report incidents or breaches in data processing. Furthermore, enabling traceability in these domains serves the purpose of demonstrating accountability, which has been recently studied extensively as part of the blockchain the literature. Traditionally, privacy and cybersecurity have been treated as distinct concepts. Even though managing cybersecurity risk contributes to managing privacy risk, it is not sufficient, since privacy risks can also arise by other means unrelated to cybersecurity incidents [9], while loss of personal data does not equate to a loss of privacy. In data terms, privacy is violated if and only if the data are used in a manner that actually violates the data subject’s fundamental right to privacy. However, as the number of privacy and data protection regulations increase, the overlap between privacy and cybersecurity increases.

Organisations are spending valuable resources by duplicating efforts to mitigate the consequences on privacy and cybersecurity attacks, competing for the same budgets. This brings us to a major challenge of having to spend a proportion of the IT budget of the organisation on countermeasures that mitigate cyber and privacy risks. This has given rise to a fairly rich literature of cyber investments seeking answers to what the best ways are to select a portfolio of countermeasures given some predefined financial limitations [10]. Within the same domain, researchers are also investigating the role of indirect costs of these countermeasures to the selection process, how countermeasures interact with each other, and what is the minimal set of countermeasures required to achieved a desirable level of overall risk.

Our work on risk assessment and control has led to the development of an innovative toolkit called AMBIENT (Automated Cyber and Privacy Risk Management Toolkit). Although AMBIENT has been designed based on end-user requirements elicited by healthcare professionals, inevitably, its functionalities can be used in other domains. Nevertheless, the knowledge bases of AMBIENT (e.g., vulnerabilities) as well as values for parameters used during risk assessment (e.g., probabilities of attack occurrence) are drawn from the healthcare domain as published in industrial reports such as the Verizon 2021 Data Breach Investigations Report.

Our motivation behind creating AMBIENT was the lack of automated software that not only conducts cyber risk assessment in the traditional way, but also takes into consideration the GDPR, healthcare processes, and then addresses the fundamental challenge of investing a financial budget to the most effective combination of cybersecurity controls. The automation nature of a cyber risk management tool is critical, because it can save time and resources of an organisation that either outsources this task or allocates a significant amount of time to combine the outcomes of the cyber and privacy risk assessments with a tool that suggests best ways to mitigate the identified risks. AMBIENT is a decision support platform that exhibits cyber risk assessment, privacy risk assessment according to GDPR terms and requirements, and cyber risk control (proactive, i.e., before threats are materialised) and mitigation (reactive, i.e., when signs of intrusions are present or new risks have been identified). At the same time, AMBIENT determines an optimal allocation of a financial budget to various cyber controls adopting the weakest link model [11].

AMBIENT is augmented with real-time intrusion detection capabilities to be able to derive changes in the risk that are worth to be considered by system administrators. Once these notifications are triggered, AMBIENT relies on Cybersecurity and a Privacy Risk Assessment modules, as solutions that take advantage of a variety of input data to perform the analysis and provide qualitative and quantitative scores that will advise organisations on the risks they are exposed to and the mitigation measures they can implement to reduce their attack surface. Such mitigation measures are shared with the Optimal Safeguard Recommendation module that performs further analysis and optimisation in order to compute prioritised list of remediation actions to be taken, acting as a holistic decision support cybersecurity toolkit.

The remainder of this paper is structured as follows: Section 2 presents the related work in cybersecurity and privacy risk assessments, as well as in optimisation of controls. Section 3 introduces the AMBIENT toolkit architecture and details its main modules. Section 4 illustrates the applicability of the proposed toolkit by analysing security threats in a healthcare infrastructure and discusses preliminary results. Section 5 discusses the paper by highlighting the main advantages and limitations of our proposed toolkit, and provides conclusions as well as perspectives for future work.

## 2. Related Work

### 2.1. Cyber Risk Assessment

An integral part of the risk assessment process is the selection of a risk assessment model or methodology. There is a vast variety of risk assessment models in the literature and tools available in the market. Examples of models used in quantitative risk assessments [12] are Fault Tree Analysis [13,14], Bayesian Networks [15,16], Monte Carlo Simulation [17], Markov Chains [18,19]. Examples of qualitative risk assessment tools are: EBIOS RM (https://www.ssi.gouv.fr/guide/ebios-risk-manager-the-method/ (accessed on 15 June 2021)) (Expression of Needs and Identification of Security Objectives); MEHARI (http://meharipedia.org/home/ (accessed on 15 June 2021)) (Harmonised Risk Analysis Method); and OCTAVE (http://www.cert.org/octave (accessed on 15 June 2021)) (Operationally Critical Threat, Asset, and Vulnerability Evaluation). IT-Grundschutz (https://www.bsi.bund.de/EN/Topics/ITGrundschutz/itgrundschutz_node.html (accessed on 15 June 2021)) (IT Baseline Protection Manual) is an example of a tool performing quantitative risk assessment. Tools such as MAGERIT (https://www.enisa.europa.eu/topics/threat-risk-management/risk-management/current-risk/risk-management-inventory/rm-ra-methods/m_magerit.html (accessed on 15 June 2021)) (Risk Analysis and Management Methodology for Information Systems) and CORAS (http://coras.sourceforge.net/ (accessed on 15 June 2021)) (a method for risk analysis of security-critical systems) are widely used for both qualitative and quantitative risk assessments. Their choice largely depends on the purpose and the data available (e.g., impact, likelihood of occurrence, etc.) [20,21,22].

Previous work by Ganin et al. [23] has intended to cover the gap between risk assessment and risk management and to allow a structured and transparent process of selecting risk management alternatives. Authors proposed a decision-analysis-based approach that quantifies threats, vulnerabilities and consequences based on multiple criteria to assess the cybersecurity risk levels. The proposed approach provides justifiable methods for selecting risk management actions consistent with stakeholder values and technical data.

Radanliev et al. [24] proposed a model for the definition of individual risks and their measurement. Authors focused on IoT scenarios and integrated an impact assessment methodology to improve understanding of the economic impact values associated with particular devices. As a result, new risk metrics were developed by considering uncertainties and potential challenges specific to the IoT environment. The major limitation of this approach is the lack of evaluation of cyber risks for the unknown but potential vulnerabilities.

Varela-Vaca et al. [25] addressed the problem of automatic security risk management by proposing a risk assessment methodology that enables the analysis and evaluation of multiple activities combined in a business process model to determine the compliance of the model with regards to the security-risk objectives. Authors focused on combining business process management and security-risk descriptions to assess the risk level of the entire process and to identify the risk responsible for a nonconformity. Artificial intelligence techniques were used to automate the presented diagnostic process.

Advances in the area of Internet of things have brought novel methods that integrate various cyber risk assessment approaches (e.g., Cyber Value at Risk [26], MicroMort [27]) to compute the economic impact of IoT cyber risks [24]. Recent cyber risk assessment models use a variety of techniques including text-mining [28], fuzzy fractional ordinary differential equations [29], Lognormal probabilistic distributions [30], among others aiming to rapidly adapt to changing environments and provide accurate risk assessment results.

### 2.2. Privacy Risk Assessment

PIA is considered as a systematic risk management approach which aims at (a) the evaluation of potential effects that systems may have on privacy [31] and (b) to foster trust by implementing the Privacy-by-Design principle [32]. Several standardisation bodies and data protection authorities have established legal frameworks and guidelines which mandate the conduction of PIA, among them the GDPR regulation [8]. However, even though the initial notion of a PIA method dates back to 2009 [31] and several published frameworks and guidelines set the principles for the conduction of privacy impact assessment, PIA remains a challenging and difficult process due to the multiple aspects that an assessor needs to consider [33]. According to GDPR, a type of processing is likely to result in a high risk to the rights and freedoms of natural persons thus the controller shall, prior to the processing, carry out an assessment of the impact of the envisaged processing operations on the protection of personal data. Nevertheless, PIA shall be an on-going process, regularly applied to personal data processing for identifying and mitigating risks in a more dynamic manner [34].

Privacy data protection standards (e.g., BS 10012:2017 [35], ISO/IEC 29151:2017 [36] and ISO/IEC 27018:2014 [37]), can be found in the literature focusing on PIA as a requirement in the execution of cybersecurity risk assessments. PIA and cybersecurity risk assessments are, however, treated as two different and uncorrelated processes [32,38], with a clear gap on automated tools, methods and models that implement PIA [33]. Even though standards (e.g., ISO/IEC 29134:2017 [39]) provide details and guidance to conduct privacy impact assessments, they are very generic, and provide high-level information that in some cases is insufficient to perform an appropriate privacy risk assessment [38]. Although the literature provides a wide variety of privacy metrics, they mainly consider properties of privacy-enhancing technologies such as the amount of sensitive information leaked or the number of indistinguishable users, instead of the privacy impact [40]. Recently, the National Institute of Standards and Technology proposed (a) a privacy framework in the form of a solid documentation and a practical tool to manage the privacy risks of an organisation by prioritising privacy protection activities through enterprise risk management [9] and (b) the NIST Privacy Risk Assessment Methodology (PRAM) that applies the risk model from NISTIR 8062 (https://www.nist.gov/privacy-framework/nistir-8062 (accessed on 7 July 2021)) and helps organisations analyse, assess, and prioritise privacy risks [41].

In addition, several national regulators have published guidelines for Data Protection Impact Assessment (DPIA), including the French Commission for Informatics and Freedom (CNIL) [42] and the British Information Commissioner’s Office (ICO) [43]. Such guidance has been updated to address GDPR’s DPIAs and to provide detailed guidelines about their regulatory requirements and processes. These guidelines follow different approaches and propose diverse steps for conducting PIA. Thus, the adoption of a single methodology becomes a difficult task for an organisation and organising a PIA project becomes a maze-like process [33]. While there are differences in the aforementioned approaches, they are equally suitable for conducting a DPIA and produce largely similar results.

The ENISA’s on-line tool [44], which consists of six steps for the calculation of the privacy risk is one of the available PIA tools. The assessment of risks is the first step towards the adoption of appropriate security measures for the protection of personal data. Furthermore, CNIL’s PIA tool [42] considers data controllers that are familiar with the PIA process. This tool lacks automation, in terms of ICT asset inventory and detection of threats or vulnerabilities that can affect privacy, that can increase the awareness of the risk assessor, while the resulted risk levels do not consider the cyber security status of the organisation. The GDPR DPIA Tool (DPIA Tool) [45] is a web-based tool for assisting organisations to evaluate data protection risks with respect to GDPR. The tool was developed to support the implementation of DPIA and provides a structured, risk-oriented approach to identification and assessment of potential data protection risks. The structure of the DPIA Tool is based on a questionnaire and thus, it offers a rather limited automation of the assessment of processing activities on personal data within the organisation. Last, the Compliance-Kit 2.0 tool [46] follows the British standard BS 10012, GDPR, and ISO 29100, and is based on the legal obligation to comply with the requirements of GDPR and management’s strategic decisiona to implement these regulations with the goal of establishing, maintaining, and developing practical and process-oriented Data Protection Management.

In addition to the aforementioned regulatory efforts, academic research has also proposed improvements to DPIA processes. These efforts include making the DPIA process more systematic and structured by proposing formal modelling techniques for privacy threats [38]. The authors in [47] proposed a comprehensive methodology for identifying data privacy risks and quantifying them, while the risk values are computed at different levels to help both senior management and operational personnel, in assessing and mitigating privacy risks. In addition, the work in [38] proposed a systematic privacy-considered information security risk assessment (pISRA) model, which can take both a privacy impact analysis and risk assessment into consideration. Finally, in [48], the authors presented an empirically evaluated privacy risk assessment framework, namely DPIA Data Wheel, based on contextual integrity, that practitioners can use to inform decision making around the privacy risks of Cyber Physical Systems (CPS). However, most of these aforementioned research efforts do not implement their proposed method/model.

### 2.3. Optimal Risk Control and Cyber Investments

Controlling cyber and privacy risks is a vital requirement for organisation to become stronger against cyber actors. This is achieved through the implementation of cyber controls, which are always coming with different types of costs, including direct ones (e.g., financial) or indirect (e.g., systems usability). Inevitably, the challenge of improving these risks not only requires ways to optimise cyber control choices, especially by combining these controls to attain greater efficacy, but poses the need for sophisticated cyber investment strategies, first studied by Gordon and Loeb [49].

The Risk Mitigation component of AMBIENT has been inspired by the work published in [50,51]. In the latter, we have published the algorithmic side of our component, including the mathematical analysis required to optimise decisions about cyber controls and cyber budget spending. These papers deploy mathematical optimisation and game theory to derive optimal strategies for the defending (e.g., the manager of the infrastructure to be protected) and attacking agents (e.g., an adversary). Applying game theory to cyber security has been proposed by several works in the literature, e.g., [52,53].

The seminal works, published by Fielder et al. [50,54], have proposed novel ways to invest a cyber security budget to protect small and medium enterprises against commodity cyber attacks. The authors have used mathematical optimisation, game theory, and cyber security engineering to assess their framework without developing a dashboard or an entire software component to offer this advice, as it is the case of the AMBIENT’s Risk Mitigation component.

In a similar vein, Wang [55] studied the tradeoffs between cybersecurity investments inn acquiring knowledge and expertise (i.e., personnel) and deploying mitigation techniques. Cyber security investments have also been studied as part of supply chain network models, where Nagurney et al. [56] empower competing retailers to maximise their expected profits through optimising product transactions as well as investments in cyber security.

A different types of work has looked into the more specific problem of when is the best time to invest in cyber security, where Chronopoulos et al. [57] proposed a real options approach to tackle this timing problem. By analysing the cost of cyber attacks and when cyber controls can be deployed by the organisation, they derive the optimal timing for such deployment optimising returns on security investments.

Besides investing in cyber controls, parts of the literature have looked into the effect of uncertainties during the risk assessment phases and how these affect the investment decisions [58,59]. The same works compute optimal strategies given these uncertainties offering cybersecurity investment models that are robust to these uncertainties meaning that they optimise return on security investment despite not having the accurate values about the probabilities of different cyber attacks being materialised.

Besides studying how to invest in a wide range of cyber controls under uncertainty, Paul and Wang [60] proposed a way to invest optimally between prevention and detection cyber controls. Dutta and Al-Shaer [61] formulate cybersecurity resilience and by considering a set of residual cyber risk, budget available to cyber controls, and finally resiliency and usability constraints propose a method to derive the best combination of critical security controls.

### 2.4. AMBIENT Novelty

In the previous sections we presented a gamut of methodologies, standards, research endeavours and tools related to the three pillars of AMBIENT namely, the Cyber and Privacy risk assessments and optimal safeguards provision. On the one hand, the cybersecurity risk assessment uses a wide base of risk indicators that analyse threat scenarios using a rule matching methodology. This approach enables the competitive advantage of the cybersecurity risk module to operate on top of detection and inventory tools for the conduction of real-time evaluation of cyber risks. In addition, both qualitative and quantitative methods are adopted for risk evaluation. The latter approach is expressed in monetary values and represents the typical loss and the worst-case scenarios to ease decision makers in perceiving the criticality of identified risks. Furthermore, based on its internal modelling, the AMBIENT’s cybersecurity module provides a list of mitigation measures that apply to each risk model to reduce risk levels.

On the other hand, the privacy module aims to bridge the gap between the cyber and privacy risk assessment, which are treated as distinct management processes [32,38]. The AMBIENT’s privacy risk assessment module operates in tandem with real-time threat inventory tools in order to quantify the privacy impact on the data processing activities of an organisation. Thus, our proposed solution exceeds the rigid approaches of privacy impact assessment by utilising an extendable scoring system and by considering the inter-dependencies of data processing ICT assets (i.e., assets which are engaged in data processing activities) to ease the privacy impact analysis on the fly. As mentioned in Section 3.2, the privacy assessment module gets advantage of inter-dependency graphs as the core modelling technique to express the connectivity and relationships between assets, data entries, threats and vulnerabilities in order to identify associated risks. Thus, the inter-dependencies assist not only on the the data flows representation, but is also used to define the processing activities and the possible attack paths for privacy risk calculation. Yet, we offer a high level of automation, which has been documented as a gap in the state of the art of PIA conduction [33]. Overall, in order to meet the requirements of the regulatory frameworks and be in line with the requirement to increase automation in supporting the PIA processes, the privacy risk assessment module of AMBIENT offers the following advantageous characteristics: (i) Asset inter-dependencies documentation, (ii) Data processing flows identification, (iii) Automation and Dynamicity of PIA, (iv) Cyber risk consideration in PIA, (v) Mitigation controls, (vi) GDPR PIA support, and (vii) Automated privacy impact scoring.

The AMBIENT’s risk mitigation module offers strategic decisions on cybersecurity countermeasures and investments based on the finding of the cybersecurity and privacy modules. The game theoretic approach of the risk mitigation component and its optimisation methodology offers decision support considering both the efficacy of the controls and the cost of implementation to infer on their optimality for mitigating the identified risks. In addition, the proposed solution takes advantage of the use of the well-documented basis of CIS controls and offers a database of tools that are recommended to the user as part of an optimal cyber strategy.

Furthermore, apart from the advancements brought by each tool individually, AMBIENT unifies the described functionalities under the same umbrella by providing a solution that can operate with a high level of automation and can support decision makers to the maze-like risk management and mitigation operations. To the best of our knowledge, AMBIENT is the only solution that offers this pipeline of tools that brings together the cyber and privacy risk assessment for real-time evaluation, and bridges the gap of responding to the identified cyber and privacy threats based on strategic investments. Through this synergy, AMBIENT can satisfy the real needs of vertical sectors that present a constantly changing cyber and privacy threat surface, such as the healthcare sector.

## 3. AMBIENT: Automated Cyber and Privacy Risk Management Toolkit

**Figure 1:**
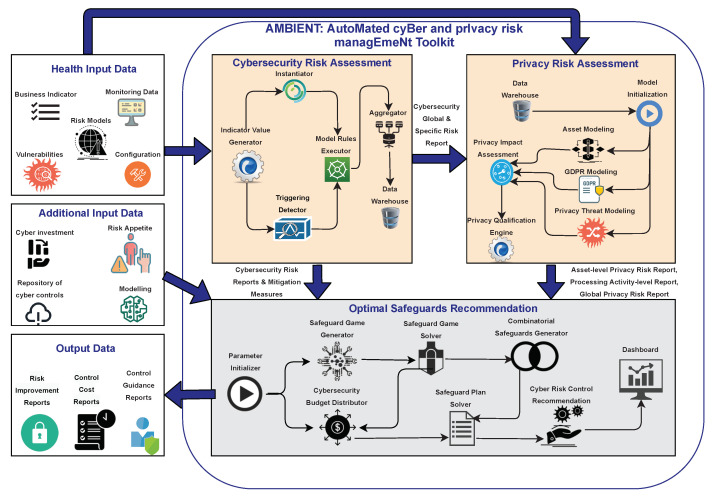
AMBIENT Architectural Model.

As depicted in Figure 1, AMBIENT is a toolkit composed of three main modules: (i) the Cybersecurity Risk Assessment, aiming to assign qualitative and quantitative risk levels to potential cyber threat scenarios; (ii) the Privacy Risk Assessment, which is responsible for analysing potential privacy threats; and (iii) the Risk Mitigation, which is responsible for evaluating, ranking and selecting optimal security measures to mitigate cyber risks.

This section describes all modules of the AMBIENT toolkit.

The toolkit receives infrastructure data (e.g., monitoring data, vulnerabilities, system configuration, risk models) and produces qualitative and quantitative risk scores that indicate the cybersecurity and privacy risk levels of an organisation, a business unit or an individual asset. Risk scores are accompanied by a list of mitigation measures that are ranked according to multiple factors and that indicates the priority to be given to their implementation.

### 3.1. Cybersecurity Risk Assessment

The Cybersecurity Risk Assessment is in charge of analysing data aggregated from multiple sources and assessing the risk level of an organisation. This module uses incident detection functionalities offering capabilities of a Security Information and Event Management (SIEM) solution [62], which can handle large volumes of security data. It produces both qualitative and quantitative cybersecurity scores of the risks which an organisation is exposed to. The cybersecurity risk assessment focuses on collecting and analysing cybersecurity events (e.g., malicious incidents) in real-time (or near-real time) and correlating them to be used as an input to the risk assessment. In addition, this module provides useful information (e.g., risk levels, potential financial losses, worst case scenarios, mitigation measures to be implemented) to help C-level managers thus improving cybersecurity awareness on Information Technology (IT) and Operational Technology (OT) related stuff. Furthermore, this module is able to capture information from cybersecurity events (e.g., detected threats, attacks, potential incidents) through the use of cyber agents deployed on the organisation’s infrastructures. The information processed by this module can be accessed by a visualisation framework that presents the main outcome in a dashboard for monitoring and response, which further helps on the generation of cybersecurity alarms and risk reports.

The CORAS tool (http://coras.sourceforge.net/ (accessed on 31 May 2021)) is used to generate graphical risk models for the risk analysis process. An important part of the risk modelling stage is to create human-readable files, which provide the graphical representation of the risk models and serve as intermediate points to create the corresponding algorithms executing such models. Each CORAS risk model uses both measurable (R (https://www.r-project.org/about.html) (accessed on 25 May 2021)) and not measurable (DEXi) [63] assessment algorithms depending on the information each algorithm provides. The programming language R is used to produce monetary risk reports for each target and risk. This algorithm is also a conversion of the risk models to R scripts to supply economic loss estimations. DEXi is used to produce qualitative overall reports using fuzzy logic and generate assessments for each target and risk within a risk model as well as the platform as a whole. The algorithm represents a qualitative adaptation of risk models to DEXi scripts which will be executed by the cybersecurity risk assessment module. Using both approaches helps AMBIENT to improve its analysis by adding complementary information about the risk level and potential consequences of a given threat if realised on the targeted system.

Besides qualitative and quantitative scores, a list of mitigation measures is provided for each risk model and if these are implemented, risk levels should be reduced to acceptable values. This list of measures is produced as a result of lookups on tables that correlate threat indicators to cyber controls. Nonetheless, AMBIENT does not enforce the mitigation measures; it is just a decision support toolkit that helps security administrators and security managers, like Chief Information Security Officers, to define mitigation strategies based on the computed scores.

Qualitative risk assessment is computed as the probability of a threat exploiting a vulnerability (i.e., Likelihood) times the consequences of such vulnerability being exploited (i.e., Impact), as typically expressed in the literature by the formula Cybersecurity_Risk=Likelihood×Impact. The latter is computed using a set of risk indicators that analyse a particular threat scenario based on a rule matching methodology. A concrete example of a quantitative risk assessment is detailed in Section 4. This module evaluates the risk level of the monitored infrastructure through the use of different algorithms whose inputs are provided in the form of indicators. These latter are built based on the values of different sources of information (e.g., vulnerability assessment tools, SIEM environments, end-user’s business profile. This module uses two main data formats: Indicators, to refer to pre-conditions taken from risk models when compared with monitored input data; and Indicator Values to refer to the real input required to compute the cybersecurity risk scores.

The Cybersecurity Risk Assessment module has the following components:

- **Indicator Value Generator:** used to collect all inputs from the external sources of information (e.g., questionnaire data, target information, vulnerabilities).
- **Triggering Detector:** receives new or updated indicator values and the target information related to the loss estimations; and the risk models selected for the assessments. The Triggering Detector invokes the Risk Model Executors upon a change in any of its inputs.
- **Instantiator:** is in charge of creating an instance (i.e., qualitative used by DEXi, and quantitative used by R) of the risk models using the current indicators values received as inputs.
- **Model Rules Executor:** performs two simultaneous analysis: (i) the qualitative risk assessment for the corresponding target and the risk model using the DEXi Model Rules Executor, and (ii) the quantitative risk assessment for the corresponding target and risk model using the Model Rules Executor.
- **Aggregator:** aims to group the individual risk assessment of an organisation per asset (e.g., workstation, server, printer, cellphone) per risk model (e.g., Denial of Service, Bypass Login, Cross-Site Request Forgery) and/or per security attribute (i.e., confidentiality, integrity, availability).
- **Data Warehouse:** represents the central data storage component, which stores the following information: (i) users and organisations (input manually by administrators); (ii) users’ configuration parameters (input by end-users); (iii) risk models; (iv) catalogues of risks, mitigation measures and indicators; (v) risk reports (results of finished risk assessment procedures); (vi) active deployed sensors; (vii) events reported by sensors; (viii) alarms reported by the Monitoring Engine; and (ix) vulnerabilities found by the vulnerability scanners.

Input and Output data processed/generated by this module are summarised in Table 1 and Table 2, respectively.

### 3.2. Privacy Risk Assessment

The Privacy Risk Assessment is in charge of assessing the privacy risk level of an organisation. This module (i) considers the current cybersecurity status acquired by the cybersecurity Risk Assessment module, and (ii) performs analysis of data processing operations to uncover potential privacy risks, in alignment with the GDPR objectives, and protect sensitive user data. More specifically, it performs privacy risk analysis based on cybersecurity evidence coming from the deployed infrastructure sensors and the documented data processing actions of the organisation. To perform this, the Privacy Risk Assessment considers the interrelations that exist among Information and Communication Technology (ICT) infrastructural assets that support data processing activities, data sources, data subjects and Personally Identifiable Information (PII) and infers the privacy risks that an organisation may face due to vulnerabilities and threats targeting it. The privacy assessment process results to a list of potential mitigation measures which could be enforced by the security administrators. These mitigation measures can only be used to help cybersecurity decision-makers (e.g., Chief Information Security Officers) to define mitigation strategies based on the computed privacy scores. The functionality of the Privacy Risk Assessment is based on two pillars, which are [34]:

- A novel and extensible *privacy risk scoring system* for quantifying the privacy risks imposed by quantitatively scaled identified vulnerabilities and threats, that have an impact on privacy when targeting assets.
- A *dynamic and extensible system model* that maps core GDPR entities and requirements for assisting the information security decision makers in keeping track of all risk-related information and assessing the degree of compliance of the organisation.

These two pillars combined, draw a competitive advantage, which is the ability to consider the cybersecurity status of an organisation and quantify the privacy risks in complete alignment with GDPR requirements.

The Privacy Risk Assessment module has the following components:

- **Data Warehouse and Model Initialisation:** They are closely related one to the other. Data Warehouse is a NoSQL database based on MongoDB (https://www.mongodb.com/ (accessed on 21 April 2021)) that stores all the external input, while the Model Initialisation component (a) “translates” the stored information, (b) consults the different modelling methods used in Privacy Risk Assessment and (c) directly feeds the corresponding components.
- **Asset Modelling:** It is based on the inter-dependency graph approach introduced in [64]. The nodes represent the individual assets and the edges represent the inter-dependencies amongst them. Such a graphical representation model is a cornerstone in the Privacy Risk Assessment, as it works as the “glue” that keeps together ICT assets, data entries, threats and vulnerabilities in order to identify risk data processing activities of an organisation. This module uses the inter-dependency types *IsConnectedTo*, *IsUsedBy*, *IsProcessedBy*, *isLocatedIn*, *isStoredOn* and *IsInstalledOn* to annotate the relation among assets. These relations are not used only to denote connections among tangible ICT assets, but also to intangible ones, such as data, health records and PIIs. Overall, by utilising the inter-dependency graphs, a security analyst is in position to identify potential privacy risks based on a cartography of assets, which encapsulate their vulnerabilities and the potential privacy threats posed against them. In this way, the inter-dependency graphs contribute, not only to the uncovering of privacy risky individual assets, but crucially, they ease in highlighting privacy risky paths which are formed by chains of assets included in a specific processing activity.
- **GDPR Modelling:** The Processing Activity is the principal aspect of the GDPR modelling that aggregates all the GDPR-related information. The main information that a Processing Activity includes can be divided in three parts: (a) the processing purpose along with the involved entities, (b) all the processed personal data assigned to specific subjects, and (c) the asset chain that is involved in the processing activity. By combining the aforementioned elements, the security analyst is in position to consolidate all the necessary information for processing activities, including the engaged supporting ICT assets, and define the dependency with intangible personal and sensitive data assets.
  Considering that information systems may store and process a huge amount of data, the GDPR modelling adopts a specific data categorisation, as the criticality of the data is not always the same. In fact, the categorisation of personal data is considered essential [65], as some processing activities may focus on publicly available data, while others on financial or even sensitive data. This indicates the need to assign a different criticality level to the aforementioned data types and treat personal and sensitive data, as data types that can clearly have a higher impact on the fundamental rights and freedoms of the individuals in case of data breaches [66]. That is, AMBIENT identifies the following categories based on the classification proposed in [67] and assigns different criticality scores according to the scoring methodology presented in [34].
  − Sensitive personal data (e.g., medical data, legal documents);
  − Personal data (e.g., data which uniquely identify a person, such as IDs, Social Security Number (SSN), personal or marital status);
  − Financial data (e.g., data related to financial transactions, accounting entries);
  − Operational data (e.g., data generated during the execution of a service, log files);
  − Other data (e.g., data that cannot be classified in any of the above categories, and belong to a lower criticality level).
  In practice it is up to the Data Protection Officer (DPO) or the security administrator to identify the correct data class when instantiating AMBIENT in the context of the identified processing activities of the organisation.
- **Privacy Threat Modelling**: This component, as its name suggests, aims to provide the threat characterisation score. Given the information of quantitatively scaled identified vulnerabilities and threats this component facilitates the privacy scoring calculation based on: (a) the type of the threat; (b) the sensitivity of the corresponding vulnerable asset; and (c) the calculated cybersecurity risk score. The aforementioned factors contribute to a formula inspired by [68], in order to reflect the impact that a cyber threat may have to the data protection and privacy dimension.
- **Privacy Impact Assessment:** The Privacy Impact Assessment component aggregates all the information from the modelling components and undertakes the calculation of the privacy scores. These scores are calculated on an asset basis and quantify the impact that a vulnerability or a threat may have due to the affected asset which is used to support data processing activities. Given the severity of the threat and the peculiarities derived from the privacy threat modelling component, the Privacy Impact Assessment component assesses the impact on fundamental rights and freedoms of the individuals, following the classification used by The European Union Agency for Cybersecurity [44]. The privacy scoring system combines two factors the threat characterisation and the privacy impact. The scoring system uses a weighted scale to focus on the impact to users’ privacy, while considering the threat. However, the exact value of the weights is a parameter that can be adjusted accordingly, given the preferences and the domain knowledge of experts in different sectors. The weighted scale formula is given by the formula Privacy_Risk=(Threat_Characterisation+2×Privacy_Impact)/3. More details on the idea behind this weighted formula can be found in [34].
- **Privacy Quantification Engine:** The Privacy Risk Quantification engine is the main component of the Privacy Impact Assessment that provides three different privacy scores: (a) the asset-level privacy score, (b) the processing activity-level privacy score, and (c) the organisation-level (global) privacy score.

It must be noted that both Cyber-Security Risk Assessment and Privacy Risk Assessment Modules consider the same cyber-security vulnerabilities identified by the same external tool (e.g., OpenVAS). However, the difference is that the Privacy Risk Assessment module categorises and prioritises the vulnerabilities by using a privacy-oriented approach based on the type of the vulnerability. For instance, the privacy score is higher when the confidentiality of data may by affected (e.g., SQL injection attack) and lower when the availability of a system is affected (e.g., DoS attack). Input and Output data processed and generated by this module are summarised in Table 3 and Table 4, respectively.

### 3.3. Risk Mitigation

Mitigating cyber and privacy risks is one of the major outcomes of information security management. This mitigation may be *preemptive* or *reactive*. By preemptively choosing cybersecurity controls, organisations reduce the likelihood of attacks exploiting their assets and causing devastating impact. The controls are acting as methods to improve the current security level of an organisation and are usually instances of well-known security frameworks such as NIST 800-53, CIS Controls, and ISO 27001 controls.

The Risk Mitigation module of AMBIENT supports organisations with mitigating risks by addressing both cyber strategic decisions (called CHANGE in Chief Information Security Officers language) and more immediate security actions (called RUN) system administrators or the security team will be benefited from implementing them. It computes optimal defensive plans of cybersecurity safeguards for decision-support, which advises cybersecurity decision-makers on how to combine various safeguards to minimise overall cyber–physical risks threatening an organisation. It comprises the Core and the Dashboard; the former implements models of cybersecurity control optimisation and cyber investment, while the Dashboard visualises the performance of the selected safeguards and the decision support guidance. The Core generates all required data to be visualised by the Dashboard. The model and mathematical frameworks used in the Core are published in [51].

The overall aim of the Risk Mitigation is to act as a decision-support tool for cybersecurity decision-makers and:

- Determine *long-term best cybersecurity strategies*, in the form of an advice, in terms of mitigating cyber and privacy risks subject to financial constraints by using fundamental principles of cybersecurity risk management to create the Core model and multi-criteria mathematical optimisation to solve the underlying decision-making challenge.
- *Visualise the cybersecurity advice* using the CIS Controls v7.1 [2], which is a well-known framework of cybersecurity safeguards, by generating practical and detailed advice on tools and processes required to implement the safeguards. It also visualises the performance of cyber controls in terms of risk mitigation.
- *Visualise the results of risk improvement* to raise awareness of decision makers on how each cybersecurity safeguard improves the security posture of the organisation by using the Dashboard.
- *Prioritise short-term cyber actions* that the organisation must take against specific cyber threats and risks identified by the cybersecurity and privacy risk modules.

The above objectives are also aligned with best practices for cybersecurity in procurement as they have been published by the European Union Agency for Cybersecurity [69]. The cybersecurity strategies consist of combinations of cybersecurity controls drawn from a well-known repository. The Risk Mitigation module uses the framework of CIS Controls, which comprises a well-known repository of cyber controls based on two items: (i) real attack data and (ii) a consensus development process, which has involved cybersecurity experts to create a prioritised list of actions that increase the cybersecurity level of an organisation mitigating both vulnerability-based and threat-based risks.

The Core consists of six components that all work together to deliver a set of Risk Control Recommendations. These offer actionable advice on what cyber controls to implement and how they perform in terms of costs and risk reduction. The advice is visualised to the user through the Dashboard. The core parts of the Risk Mitigation module are:

- **Risk Parameters Initialiser**: this component initialises the parameters required to compute the optimal set of safeguards that mitigate cyber and privacy risks. They include the reports received by the risk assessment modules.
- **Safeguard Game Generator**: this component uses AI optimisation generating a *strategic game* between a defending and an attacking agent based on the game-theoretic concepts used to compute equilibria, i.e., optimal points [70]. This game is represented by the available actions of the agents and their payoff functions. The defending agent can choose among different ways of implementing a cyber control and the attacking agent among different attack methods.
- **Safeguard Game Solver**: this component calculates the game equilibria, which are optimal combinations of implementation ways (can be seen as levels when the ways refer to different intensity of implementing the control) chosen for each control used to mitigate cyber and privacy risks against the attacking agent. The Risk Mitigation module uses the 20 CIS Controls, which include 171 sub-controls. This component solves the game for each of these subcontrols to create a repository of optimal available safeguards.
- **Cybersecurity Budget Distributor**: this component takes a budget and distributes it among all the 20 CIS Controls, which is then allocated to its safeguards.
- **Combinatorial Safeguards Generator**: for each CIS control, this component generates all the combinations of safeguards, which have been calculated previously by the Safeguard Game Solver.
- **Safeguards Plan Solver**: for the budget allocation derived previously, this component calculates the safeguard combination that fits into the available budget of the defending agent and minimises the maximum risk inflicted by the attacking agent respecting the “weakest link” concept [11].

The Dashboard is used for three main high-level purposes: to communicate to the user the optimal Risk Control recommendation; to visualise its performance, in terms of risk reduction; and to visualise the different costs, direct and indirect, of the safeguards included in the recommendation.

Input and Output data processed and generated by this module are summarised in Table 5 and Table 6, respectively.

## 4. AMBIENT Demonstration

This section presents one threat scenario composed of two attacks: an SQL injection and a Ransomware attack, both mitigated by AMBIENT. The scenario evaluates concerns with the cybersecurity and privacy awareness level of the organisation regarding data exchange in remote healthcare services as inspired by the use cases of the H2020 CUREX project [71]. Cybersecurity and privacy risk assessments are conducted by the Cyber Risk Assessment and Privacy Risk Assessment components of AMBIENT and then optimal security measures are proposed by the Risk Mitigation component.

Healthcare Point of Care (PoC) systems have been widely used in hospitals in order to provide innovative solutions to medical professionals and physicians and provide them with an overview of the patients’ condition in a way that it makes easier for them to respond on time and prevent critical situations. POC systems are platforms that incorporate devices and applications in order to collect, process and visualise data. Naturally, these types of platforms expose an expanded attack surface, as the variety of devices and systems used have unique vulnerabilities, which can be challenging to identify and address. With an ever-increasing amount of data, which contain personally identifiable information and sensitive medical data, being communicated across various devices, back-end analytic platforms, and user workstations, smartphones or sensors, it becomes evident that there are multiple threats that can breach legitimate systems and data. Hospitals and care centres need to address such cyber–physical challenges by efficiently assessing the associated risks and mitigate them with appropriate cybersecurity safeguards.

### 4.1. Testbed Description

**Figure 2:**
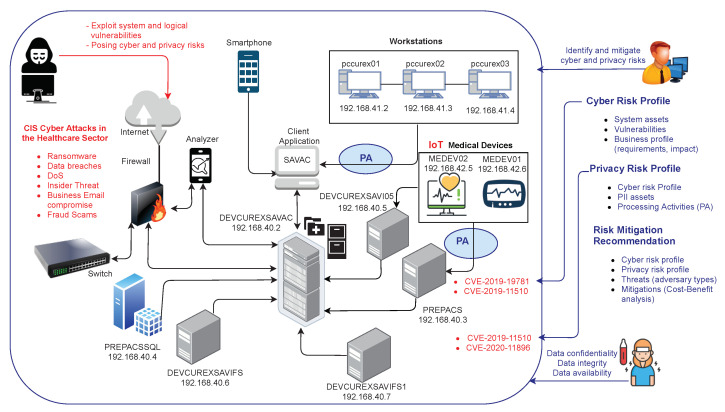
Hospital Testbed Scenario.

As depicted in Figure 2, and for the purpose of demonstrating the competitive advantages of AMBIENT, we have considered a real test-bed composed of a subset of the assets and devices used in the Spanish Hospital Fundació Privada Hospital Asil de Granollers (FPHAG) (http://www.fphag.cat/ (accessed on 31 May 2021)), which includes the following elements:

- SAVAC client application: either accessed from the CITRIX (https://www.citrix.com/ (accessed on 17 April 2021)) server farm or from a PC that has the client version installed locally, connects to the SAVAC database, which is installed in the hospital’s Data Center (DC).
- Workstations: 3 PCs are placed inside the users’ VLAN with the basic programs together with hospital’s user credential handling procedures.
- SAVAC Database server: consists of a cluster of servers that contains all the information stored.
- Firewall: The hospital’s DC is generally supervised by a firewall system in which specific rules are programmed. Virtual LAN users, servers and devices are bi-bidirectionally connected to the hospital’s firewall.
- Switch: This network component has a dedicated link to the cluster of servers and another to the rest of the network’s elements.
- Analyser: The hospital uses analytic platforms, which operates directly on data collected by SAVAC and generate analytic dashboards and visualisation reports for the hospital managers.
- PACS image server: The images are stored on a server called PACS (Picture and Archiving Communications System). To retrieve the images, a call is made from SAVAC to a URL through a unique identifier of the patient’s image study.
- Integration server: It is used to collect all data and external files and integrate them into SAVAC, either in the database or on the file server, or by external links using identifiers, as in the example of the PACS image server.
- Medical equipment: Different medical devices are placed in VLAN.
- Smartphone: Smart devices connect through hospital’s Wi-Fi (open Wi-Fi validated via capture portal), using a specific identification that the hospital’s firewall allows to be visible, to operate and to have internet access.

In the test-bed infrastructure depicted in Figure 2, the PCs run under Windows 10 operating system; the servers are placed in a VMware and the medical devices will run in different versions of Windows and Linux operating systems. In addition, the mobile application to be assessed will run on an Android v9 smartphone. More information about the testbed and use case scenarios can be found in [72].

### 4.2. Use Case Scenarios

AMBIENT has been designed to be used in the healthcare domain for a variety of use case scenarios related to health data exchange. Examples of use case scenarios where the toolkit can be useful include the following:

- Cross-border patient data exchange, originated when a patient from hospital A has a malaise in a foreign hospital (i.e., hospital B) and due to the emergency hospital B requests the patient’s health records to hospital A.
- Data exchange in mobile healthcare platforms, which considers malfunctioning of IoT devices that fail to register measurements which imposes not only service disruption but also threats to compromise the patients’ safety and health.
- Data exchange in remote healthcare services, which includes threats related to the confidentiality and integrity of the patients’ data, coming from healthcare devices and applications (e.g., mobile applications for collecting blood pressure, health rate, temperature, etc.).
- Data exchange for healthcare research, which includes privacy challenges originated from the exchange of health data for research purposes with third parties such as universities and research groups.

In addition to health data exchange scenarios, AMBIENT is able to analyse a wide variety of threats affecting the appropriate operations of healthcare organisations. Such threat scenarios are derived from risk patterns that include inputs related to different vulnerabilities, incidents and/or infrastructure context that may cause an undesirable situation with a certain likelihood, and which may have consequences in the risk level and economic loss in terms of confidentiality, integrity and availability. Examples of these risk patterns are: Denial of Service Attack, Invalidated Redirects and Forwards, Bypass Login, Compromise security via Trojan-malware, Client-Server Protocol Manipulation, Session Fixation, Cross Site Request Forgery, SQL Injection, etc.

### 4.3. CVE & Threat Model Selection

A vulnerability analysis performed in the target infrastructure identified a list of Common Vulnerability and Exposures (CVEs (https://cve.mitre.org/) (accessed on 21 June 2021)), from which the hospital security team decided to analyse those with critical severity (i.e., with a Common Vulnerability Scoring System –CVSS (https://www.first.org/cvss/ (accessed on 21 June 2021))– higher or equal to 9.0).

As a result, two critical vulnerabilities have been identified in some of the hospital’s assets, the exploitation of which could directly affect the IoT medical devices (i.e., MEDEV01 and MEDEV02):

- CVE-2020-11896 (https://cve.mitre.org/cgi-bin/cvename.cgi?name=CVE-2020-11896 (accessed on 31 May 2021)) which allows remote code execution related to IPv4 tunneling;
- CVE-2019-11510 (https://cve.mitre.org/cgi-bin/cvename.cgi?name=CVE-2019-11510 (accessed on 31 May 2021)) which allows attackers to remotely access the targeted network and perform arbitrary file reading.

The hospital’s IT department has raised concerns about the cybersecurity and privacy issues that may emerge from the operation and the communication of the clinical data. Since the data contain highly sensitive personally identifiable information, it must be ensured that the hospitals’ information systems are properly maintained, and any vulnerabilities are identified and timely patched. In addition, since the hospital has the technical capability of generating data reports and exchanging them with third parties, the platform must ensure that proper cybersecurity and privacy safeguards are in place in order to protect the integrity of the data and most importantly the patients’ safety. Consequently, the hospital integrates AMBIENT to perform a cyber and privacy risk analysis in order to immediately address risks that exceed the acceptable levels.

Two threat scenarios have been associated to the critical vulnerabilities found in the hospital: an SQL injection (exploiting CVE-2020-11896 via a remote code execution), and a Ransomware attack (exploiting CVE-2019-11510). The main concern about an SQL injection attack is that it could allow the intruder to change, delete and add patients and hospital information and cause malfunctions on the regular procedures. A security compromise via Ransomware is a user level threat, which can potentially give access to an intruder to the forms and tests of patients stored in the hospital servers. A negative concern about this threat is the possibility to allow the intruder to encrypt a vast part of the patients and hospital information processed by the medical devices (e.g., MEDEV01, MEDEV02) and ask for a payment to rescue the information and/or consequently stop most of the hospital ongoing activities. These types of attacks can have a greater impact on users’ privacy, since the attacker can have direct access to the sensitive data, in contrast to Denial of Service (DoS) attacks that mainly affect the availability of the asset.

### 4.4. Cybersecurity and Privacy Risk Assessment Results

The cybersecurity and privacy risk modules receive this information along with a list of events and alarms detected in the target system. The identified threat is directly affecting all workstations from the network 192.168.41.0/24, as well as the PREPACSQL server and the SAVAC servers (DEVCUREXSAVAC, DEVCUREXSAVI05, DEVCUREXSAVIFS, and DEVCURESSAVIFS1). Both modules perform their risk analysis and generate individual risk scores considering cyber and privacy issues. The JavaScript Object Notation (JSON) [73] file shown in Listing 1 corresponds to the cybersecurity risk assessment output generated for this evaluation.

Listing 1 **Cybersecurity Risk Assessment Report for a combined attack.**

{‘id’: 116144,

‘report’: 0001,

‘risk_model’: WPR4, WPR8

‘risk’: ‘C’, ‘I’, ‘A’,

‘target’: ‘192.168.41.2’, ‘192.168.41.3’, ‘192.168.41.4’, ‘192.168.40.2’, ‘192.168.40.4’,

‘192.168.40.5’, ‘192.168.40.6’, ‘192.168.40.7’,

‘qualitative_assessment’: ‘VH’,

‘quantitative_assessment’: ‘25320.69:45818.00’,

‘mitigation_measures’: [‘M8’, ‘M10’, ‘M13’, ‘M18’, ‘M19’, ‘M22’, ‘M41’, ‘M42’, ‘M43’, ‘M44’,

‘M45’, ‘M46’, ‘M47’, ‘M48’, ‘M49’ ],

‘timestamp’: ‘2020-01-09T19:22:18.222345Z’}

**Figure 3:**
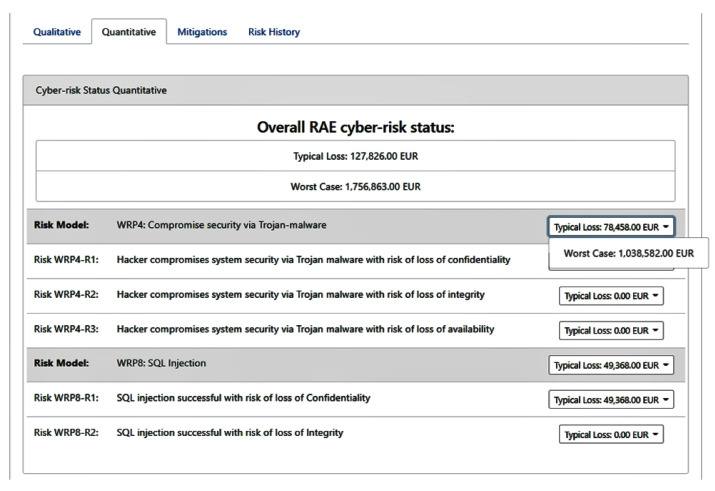
Cybersecurity Quantitative Risk Score.

The previous report indicates that the risk model WPR4 that corresponds to a security compromise via trojan-malware (i.e., Ransomware) and the risk model WPR8 that corresponds to an SQL injection, are assessed as high and very high, respectively, with potential damages that could range from 127,826 EUR to 1,756,863 EUR (as depicted in Figure 3). For these threats, we have identified a set of mitigation measures and we have assessed their efficacy and cost in this scenario, based on the expert knowledge of the end-user’s team.

Similarly, the privacy risk module generates the output of the privacy assessment process as depicted in Listing 2. As already mentioned, three different privacy risks scores are generated, namely the privacy score per asset, per processing activity and the global privacy score of the organisation. The global privacy score is based on the maximum of scores of the processing activities, while the score of a processing activity is the maximum of the included assets. The representation of the aforementioned outputs would result in a lengthy technical documentation that goes beyond the scope of this paper.

**Figure 4:**
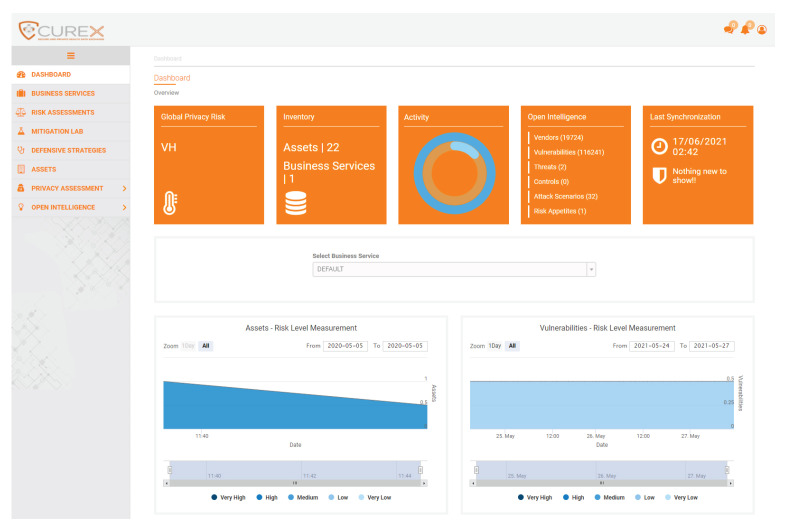
Privacy Risk Assessment Dashboard.

Listing 2 indicates the privacy risk analysis performed by the privacy risk assessment tool over the potential SQL injection and ransomware attacks. The risk level assigned to the organisation is a product of the analysis performed on the defined data processing activities of the organisation given the set of the supporting assets affected by the SQL injection and Ransomware attack. Figure 4 displays the main dashboard with the calculated global privacy score and some additional statistics. From the qualitative perspective, it assigns a Very high level of risk, while 9.8 out of 10 for the quantitative one. The privacy risk is calculated considering the peculiarities of each case, i.e., the sensitivity of the asset, the vulnerability type, the type of processed data and the number of the affected processing activities. More details regarding the quantification methodology and formula can also be found in [34]. Given the great magnitude of an SQL injection against the PREPACSQL asset, which is used to store and process sensitive personal information of patients, the privacy risk model considers the inter-dependencies and derive the aforementioned privacy risk level.

Listing 2 **Privacy Risk Assessment Report for a combined attack.**

{ ‘global_privacy_score’: {

   ‘riskassessmentId’: 117,

   ‘organization’: ‘FPHAG’,

   ‘privacy_quantitative’: 9.8,

   ‘privacy_qualitative’: ‘VH’},

‘timestamp’: ‘2020-01-09T09:46:15.242445Z’}

The joint risk computed from the cybersecurity and privacy results in a VERY HIGH level, which requires the implementation of security measures before undertaking any data exchange with third-party organisations.

### 4.5. Risk Mitigation Results

This section details information about reactive and preemptive controls used by the risk mitigation module of AMBIENT to select optimal mitigation strategies against the analysed threat scenario.

#### 4.5.1. Reactive Controls

For this threat scenario, we have identified a set of mitigation measures and have assessed their efficacy and cost based on the expert knowledge of the end-user’s team. Table 7 shows the suggested countermeasures to address the identified risks. We have used the Risk Mitigation component of AMBIENT to prioritise the list of remediation actions given that each of them has a benefit and a cost. This component can also be used to provide a cyber strategy of the organisation given the aggregated risks identified by the cybersecurity and privacy risk assessment modules and offering preventative cyber and privacy risk reduction capabilities.

Based on the information associated to each mitigation measure, the risk mitigation module generates the JSON file depicted in Listing 3 as an output of its analysis.

Listing 3 **Risk Mitigation Report for an SQL injection attack.**

{ ‘risk_mitigation_output’: {

‘id’: 0101,

‘report’: 0001},

‘mitigation_measures’: [{‘M49’,‘M8’,‘M22’,‘M41’,‘M42’,‘M13’,‘M44’,‘M10’,‘M45’,‘M43’,‘M46’,

‘M19’,‘M18’,‘M48’}],

‘timestamp’: ‘2020-01-09T09:46:15.242445Z’}

#### 4.5.2. Preemptive Controls SUBJECT to a Budget

**Figure 5:**
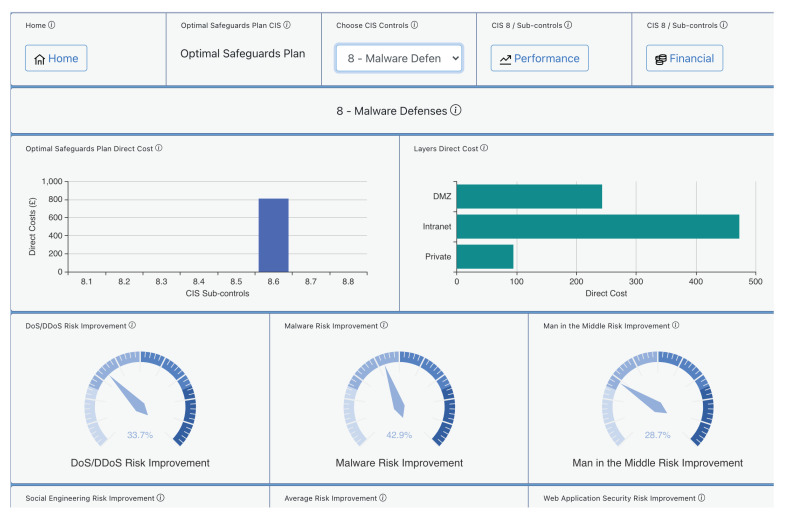
Risk Mitigation output for the CIS 8 “Control Malware Defences”.

Given a specific cybersecurity investment budget, AMBIENT can derive the best combination of cyber controls by using the risk mitigation module. This latter is run for a budget of EUR 20,000 to be spent on implementing CIS Controls. Figure 5 provides examples of the risk mitigation output data for the CIS Control 8 “Malware Defences”. We notice that the tool has selected the sub-control “Centralise Anti-Malware Logging”, which implements the capability of “sending all malware detection events to enterprise anti-malware administration tools and event log servers for analysis and alerting”.

The advice suggests that by implementing this sub-control the organisation will benefit from improvement in various different areas of threat prevention such as DoS, Malware, and Man-in-the-Middle attacks. The direct cost is also presented there split into the different network layers (Demilitarized Zone-DMZ, Intranet, Private Subnet) for this specific sub-control in this organisation.

**Figure 6:**
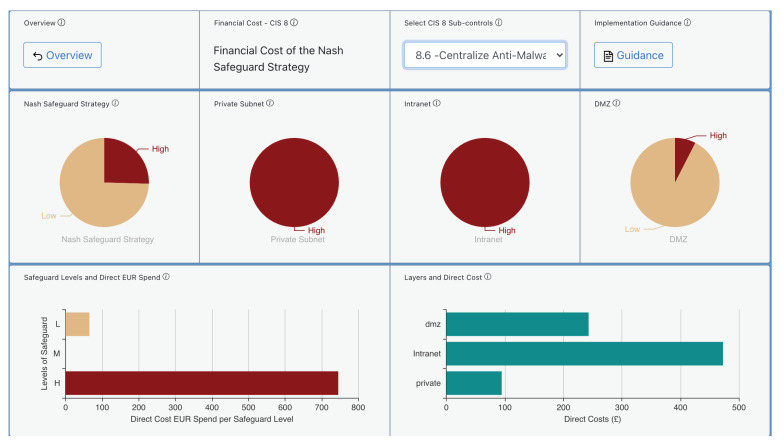
Risk Mitigation Dashboard Output.

Besides, Figure 6 provides examples of the risk mitigation output data presenting the cost levels for the proposed CIS 8 Control “Malware Defences” using 8.6 “Centralise Anti-Malware Logging”.

## 5. Discussion and Conclusions

### 5.1. Discussion

Most risk assessment tools found in the literature either perform a cybersecurity or a privacy risk assessment. Most tools on the market have a rather narrow scope of application, with a single use case being the norm. There exists a considerable number of tools supporting the documentation of data processing practices, the formulation of consent templates, or the documentation of privacy and data protection policies. Apart from CNIL’s PIA tool, available methods make no reference to any tools that can automate the PIA process or create a PIA report. While, the cyber security status of the organisation in which the impact analysis is performed is largely neglected by those tools.

Furthermore, the current state-of-practice on risk assessment and analysis of data-related vulnerabilities in the healthcare domain includes mostly custom and proprietary solutions (tools, mechanisms, techniques, procedures) that are typically employed on demand, e.g., when new systems or components are installed, or when new policies need to be enforced. Most standards specify framework conditions for the risk management process but rarely go into detail of specific methods for the risk analysis or risk assessment. This is one of the reasons why often differences in the risk assessment arise within specific areas of application, such as in healthcare, making a direct comparison of the results cumbersome.

In addition, the interoperability and information sharing among the underlying security-related components (usually coming from different technology providers and vendors) is hard to achieve, which in turn increases operational costs and stress during the daily tasks in hospitals and care centres. As a consequence, the healthcare sector is still far from a unified framework that will address vulnerability and risk assessments and analysis through a holistic solution that will compose and orchestrate the heterogeneous tools, mechanisms, techniques, procedures, while respecting privacy and adhering to GDPR policies.

Regarding limitations of the toolkit, in order to perform the cybersecurity risk assessment, AMBIENT requires a minimum set of input data from the healthcare organisation. If no vulnerability is detected or no business profile is provided by the healthcare organisation, our proposed toolkit will not be able to compute the cybersecurity risk score. The qualitative and quantitative risk scores associated with the SQL injection attack discussed in Section 4 evaluates a threat scenarios that considers eight indicators (see Table 8) as well as the identification of potential vulnerabilities and weaknesses. The exploitation of these weaknesses will potentially lead to four unwanted incidents (i.e., privilege escalation by an attacker, unauthorised reading of data, unauthorised code execution, unauthorised modification of data). Such incidents, if realised, will negatively impact confidentiality, integrity, availability of the infrastructure’s assets and hence privacy.

Limited input data will lead to inaccurate cybersecurity and/or privacy risk scores which will affect the selection of optimal mitigation measures. Cybersecurity and privacy scores are therefore highly dependent on the input data provided by the target infrastructure. Similarly, the risk mitigation module highly depends on the input data provided by the cybersecurity and privacy modules about the potential mitigation measures for each threat scenario.

In addition, it is important to mention that AMBIENT is a decision support tool that provides advise and guidelines to security analysts and administrators on the basis of the potential risks affecting their infrastructures, but it does not implement automatic security actions to reduce the organisation’s risk level. Security administrators and C-level managers should decide which strategy to deploy, and use the AMBIENT outcome as a guide in their decision-making process. The implementation of a mitigation measure therefore requires manual intervention from the healthcare infrastructure and will generate an effect on the cyber climate, as it will change indicator values in the system, and consequently, cybersecurity and privacy risk scores are expected to decrease. Mitigation measures are displayed in a simple way in the toolkit dashboard (including an ID, the name and a description of the mitigation measure), and can also be shared in the JSON format.

Another important aspect in the risk assessment process is the time spent for AMBIENT to compute their results. In general, scores are generated within a few minutes (depending on the type of threat analysed and the number of indicators to compute), and mitigation measures are analysed within seconds (right after the safeguard candidates are generated by the cybersecurity and privacy modules). The cost/benefit information associated to each mitigation measure is an input data obtained through expert knowledge from the healthcare technical personnel. The accuracy of the results provided by risk mitigation module depends on the reliability of the provided input data, and the statistical and mathematical model used in the evaluation. It is worth noting that the risk mitigation part of AMBIENT is able to analyse not only reactive mitigation measures but also proactive mitigation measures. The former are immediate actions required to eliminate or reduce the risk level of a given organisation, whereas the latter refers to medium or long-term actions required to reduce the attack surface of the target organisation. The two threat scenarios presented in Section 4 only include reactive mitigation measures, as we have assumed that the risk assessment is requested after a security event has been detected on the system. AMBIENT relies on this technology to enforce GDPR and record the risk assessment reports. As a result, the proposed approach will control the health data exchange process by reinforcing security, improving traceability and auditability functionalities.

### 5.2. Conclusions

This paper described AMBIENT (Automated Cyber and Privacy Risk Management Toolkit) that evaluates and analyses the cyber and privacy risks of an organisation and recommends mitigation measures that maximise risk reduction given a knowledge base of countermeasures, along with their direct and indirect costs, and subject to a financial budget. The proposed toolkit is composed of three main modules: a Cybersecurity Risk Assessment module, responsible for analysing potential cyber threat scenarios; a Privacy Risk Assessment module, responsible for analysing potential privacy risks in alignment with the GDPR objectives; and a Risk Mitigation module responsible for evaluating and selecting optimal measures to mitigate selected risks.

AMBIENT was deployed in a healthcare infrastructure to evaluate different attack scenarios potentially affecting their daily operations. As such, AMBIENT has been created to support cybersecurity decision makers with cybersecurity and privacy assessment identifying critical assets, potential threats to face, consequences that these threats may cause if they occur, and the actions to be implemented for their mitigation.

As a decision support tool, AMBIENT provides advise on the basis of the potential security and privacy risks affecting target infrastructures, however, it does not automatically implement mitigation actions. Security managers can use AMBIENT’s results as a guide in their decision-making process to define appropriate security policies and strategies that keep risk scores within acceptable levels. Future work will focus on the integration of additional threat and vulnerability frameworks towards a more holistic risk management suite of tools. Additionally, exploring novel privacy-preserving techniques in the context of blockchain applications is left as future work which could bring more possibilities to extend the use of AMBIENT. It will also investigate the applicability of the toolkit to mitigate social attacks, which represent the highest risk to organisations and appear in the most claims as reported by cyber insurers.

## Figures and Tables

**Table 1:** Input to the Cybersecurity Risk Assessment Module.

Input Data	Description	Expected Data
**Business indicators**	Questionnaire about the organisation’s size, business structure and main security aspects as well as information about the economic impact of the organisation and details on the confidentiality, availability and integrity affecting values.	Q14. Do browsers used in your organisation allow client side scripting? Yes = 1, No = 0, Do not know = 0.
**Monitoring data**	Information of the monitoring infrastructure in the form of events (e.g., expiration of a license key, a virtual machine is powered on, user logs on a virtual machine, fail login requests, host connection lost) and/or alarms (e.g., detected malware, Man in the Middle attack, potential brute-force attack, connection attempts against SQL services). They include details of the source elements (i.e., IP address, port number, detection device), as well as details on the time associated to the event and the type of event detected.	- SQL injection attempts detected, 3 events, severity(2) low, src 91.189.88.152:3510, dst 192.168.40.4:41814 - Malware detected, 2 events, severity(6) medium, src 199.232.150.232: ANY, dst 192.168.40.2:46976.
**Vulnerabilities**	Contains the list of potential vulnerabilities affecting the target infrastructure. They are compared against the indicator rules to get inputs for the algorithms used in the risk models. New detected vulnerabilities will automatically trigger a new risk assessment.	CVE-2020-11896, CVE-2020-11903, CVE-2020-11914, CVE-2019-11510.
**Configuration data**	Changes or updates in the *configuration* of the target infrastructure (e.g., IP addresses and ports of the available machines, addition or removal of assets, estimation of the confidentiality, integrity or availability impact).	MEDEV02: C = 9, I = 7, A = 5, PREPACSSQL: C = 10, I = 10, A = 10.
**Modelling**	Refer to the selected risk models that are pre-defined and associated with specific algorithms (script files). The toolkit uses these models and rules to compare with real input in order to represent a situation inside a risk model.	WPR4: Compromise security via Trojan malware, WPR8: SQL injection.

**Table 2:** Output Generated by the Cybersecurity Risk Assessment Module.

Output Data	Description	Expected Data
**Cybersecurity Global Risk Reports**	Qualitative and quantitative global risk scores indicating the *overall risk* associated to the target organisation. Global qualitative risks range from Very Low to Very High, whereas global quantitative risks are expressed in monetary values and represent the typical loss and the worst-case scenario.	“risk model”: “WPR8”, “target”: “FPHAG”, “cyber qualitative”: “VH”, “cyber quantitative”: “49368: 721267”
**Cybersecurity Specific Risk Reports**	Cybersecurity reports indicating qualitative and quantitative assessment associated to the analysed threat per model (e.g., DoS, Malware, Bypass Login, SQL injection, etc.), per target asset (e.g., workstation, server, medical device, etc.), and per risk (confidentiality, integrity, availability.	“risk model”: “WPR8”, “risk”: “C, I”, “target”: “SQL Server (192.168.40.4)”, “cyber qualitative”: “M”, “cyber quantitative”: “9928: 145298”
**Mitigation Measures**	List of mitigation measures associated to the analysed threat and that are proposed to be implemented by the end-users to eliminate or reduce the risk down to acceptable levels. The selection of these mitigation measures is further processed and analysed by the Risk Mitigation module of AMBIENT.	“risk model”: “WPR8”, “target”: “FPHAG”, “measures”: “M8, M10, M13, M22, M41, M42, M43, M45, M46, M47, M48, M49”

**Table 3:** Input to the Privacy Risk Assessment Module.

Input Data	Description	Expected Data
**Monitoring Data, Vulnerabilities and Cybersecurity risk**	Information acquired from the monitored infrastructure, including the identified vulnerabilities, as a result of external asset inventory, network scanning tools (e.g., Open Vulnerability Assessment Scanner - OpenVAS (https://www.openvas.org/) (accessed on 12 July 2021)) and cybersecurity risk tools.	**Monitoring data:** src 91.189.88.152:3510, dst 192.168.40.4:41814 **Vulnerabilities:** CVE-2020-11896, CVE-2020-11903, CVE-2020-11914, CVE-2019-11510 **Cybersecurity risk:** “risk model”: “WPR8”, “risk”: “C, I”, “target”: “SQL Server (192.168.40.4)”, “qualitative”: “Medium”, “quantitative”: “9928:145298”
**Configuration data**	This input is provided by end-users and reflects the infrastructure profile and environment. More specifically, this type of information could be, among others, the Personal Data to be processed, the Data Subjects (e.g., Patient), the Legal Grounds, the Legal Entities, the Processing Types, the Processing Activities, the Attack/Threat Scenarios, the privacy-oriented value of assets and already applied mitigation controls.	**Configuration data: **“PIIs”: “name, surname”, “data subjects”: “Patient1”, “legal entity”: “DPO”, “legal grounds”: “Legal monitoring of Patient1”, “type”: “transfer health data”, “activities”: “91.189.88.152, 192.168.40.4”, “privacy value”: “CVE-2020-11896:VH”

**Table 4:** Output Generated by the Privacy Risk Assessment Module.

Output Data	Description	Expected Data
**Asset-level Privacy Risk Report**	It is a privacy score associated with a specific asset that faces a possible privacy threat. Thus, each asset has a threat characterisation score associated with a privacy impact score.	**asset1:** “asset”: “192.168.40.4”, “privacy quantitative”: “5.0”, “privacy qualitative”: “M”, **asset2:** “asset”: “91.189.88.152”, “privacy quantitative”: “9.8” “privacy qualitative”: “VH”
**Processing Activity-level Privacy Risk Report**	It is a privacy score associated with a data processing activity. A processing activity may consist of several assets. Thus, the risk level of a processing activity is the maximum value of risk among the assets participating in the processing activity.	**processing activity1:** “processing activity”: “91.189.88.152, 192.168.40.4” “privacy quantitative”: “9.8” “privacy qualitative”: “VH”
**Global Privacy Risk Report**	It is a privacy score associated with the whole organisation. The risk score will be the maximum risk among the processing activities. Note that, the global privacy score is combined with the risk score derived from the cyber security assessment module.	**global:** “privacy quantitative”: “9.8” “privacy qualitative”: “VH”

**Table 5:** Input to the Risk Mitigation Module.

Input Data	Description	Expected Data
**Cyber investment**	The budget available to the defending agent to implement cyber controls.	“Available budget”: “30,000 EUR”
**Cyber and privacy risk reports**	The outputs of the other two modules of AMBIENT, used by the Risk Mitigation module to decide on how to mitigate risks using controls.	“risk model”: “WPR8” “measures”: “M8, M10, M13, M22, M41, M42, M43, M45, M46, M47, M48, M49” “privacy quantitative”: “9.8”, “privacy qualitative”: “VH”
**Risk appetite**	The organisation chooses its own risk appetite expressed in the degree of impact they can tolerate before they decide to spend a greater cybersecurity budget.	“risk appetite”: “Medium”.
**Repository of cyber controls**	This requires a repository of controls along with their costs (purchase, implementation, maintenance cost) and benefits (efficacy in mitigating threats) to evaluate them during the game-theoretic and the optimisation phase of its operation.	“CIS subControls”: 1.1: “directcost”: 2276.158891, “implementation level”: {H: { “implelevel”: 1, “system performance cost”: 7.491514705, “usability cost”: 6.493688798, “overall indirectcost”: 6.992601751, “efficacy”: 78.36633331, “directcost”: 4097.086004 },

**Table 6:** Output Generated by the Risk Mitigation Module.

Output Data	Description	Expected Data
**Reactive Risk Mitigation Report**	This report shows which mitigation measures, as proposed by the cyber risk assessment module, must be used and with what priority optimised by the risk mitigation module.	“risk mitigation output”: ’id’: 0101, ’report’: 0001, “mitigation measures”: M49, M8, M22, M41, M42, M13, M44, M10, M45, M43, M46, M19, M18, M48, “timestamp”: 2020-01-09T09:46:15.242445Z.
**Risk Improvement Reports**	These demonstrate to the end-user the degree of risk improvement when the proposed safeguards are chosen for implementation. Each selected safeguard exhibits its own improvement against different threats. This is key in allowing the end user to make an informative choice about the required preemptive controls.	‘1’: { “malware risk improvement”: 99.11941836, “dos risk improvement”: 96.70459599, “web attack improvement”: 99.18107606, “phishing risk improvement”: 96.01828912, “man in the middle risk improvement”: 99.40160608, “overall risk improvement”: 98.72331726, ...}
**Control Guidance Reports**	These are reports that explain to the end-user how the selected safeguards assist the organisation. They also include suggestions about actual cybersecurity products mapped to the framework of controls selected.	Information about the proposed CIS controls with suggestions on how they will help the target organisation ** (as presented in Figure 5 and Figure 6).
**Control Cost Reports**	These reports show to the end-user what costs must be tolerated if the proposed safeguards are selected. The costs refer to both *direct* (e.g., financial losses) and *indirect* (e.g., system performance loss).	“CIS Sub-control”: 1.1, “Implementation Level”: Low, “System Performance Cost”: 0.966163655, “Financial Cost”: 1.789499785, “Usability Cost”: 0.386993268

** For this we have used the document published by the US Department of Homeland Security and Emergency Services, Cyber Incident Response Team.

**Table 7:** Mitigation Measures for SQL injection and Ransomware against the target Hospital.

MM	Description
M8	Validate input
M10	Map input values to actual filenames/URLs and reject all other input
M13	Use application firewall to detect attacks against URL redirection
M18	Use antivirus software that is currently considered to be strong by experts in the field
M19	Verify the integrity of the software that is being installed
M22	Ensure checks performed at the client side are duplicated on the server side
M41	Use vetted library to mitigate improper neutralisation of special elements used
M42	Use structured mechanisms to enforce automatic separation of data and code
M43	Run code using the lowest privileges to accomplish the necessary tasks
M44	Quote arguments and escape any special characters within dynamically generated queries that mix control and data together
M45	Ensure error messages contain minimal details useful only to the intended audience
M46	Avoid using register global in the application
M47	Mix white and black list parsing to filter control-plane syntax from input
M48	Handle exceptions at code level
M49	Fix errors returned by functions

**Table 8:** Risk Indicators for an SQL injection attack.

Indicator	Means	Data-Type	Source-Type
IN-32: Does the web application consist of HTML forms?	questionnaire	Boolean	business
IN-37: Do HTTP requests contain special elements used in an SQL command successfully executed?	event	Boolean	test
IN-38: Do records in the database consist of corrupt or invalid data?	vulnerability	Boolean	application
IN-44: Do HTTP requests contain special elements used in an SQL command?	event	Boolean	network
IN-45:Do HTTP responses contain malicious scripts?	network, test, app	Boolean	network
IN-54: How many sanitised HTTP requests contain special elements used in an SQL command?	application	Integer	event
IN-55: How many abnormal (suspicious) SQL queries are executed?	application	Integer	event
IN-56: How many SQL-related errors have been recorded in the log?	application	Integer	event

## Data Availability

Not applicable.
